# Effects of combined decision-support and performance-based incentives on reported client satisfaction with maternal health services in primary facilities: A quasi-experimental study in the Upper East Region of Ghana

**DOI:** 10.1371/journal.pone.0249778

**Published:** 2021-04-20

**Authors:** Gifty Apiung Aninanya, Easmon Otupiri, Natasha Howard

**Affiliations:** 1 Department of Health Services Policy, Planning, Management and Economics, School of Public Health, University for Development Studies, Tamale, Ghana; 2 College of Health Sciences, Kwame Nkrumah University of Science and Technology, Kumasi, Ghana; 3 National University of Singapore, Saw Swee Hock School of Public Health, Singapore, Singapore; 4 London School of Hygiene and Tropical Medicine, Department of Global Health and Development, London, United Kingdom; Erasmus Medical Center, NETHERLANDS

## Abstract

**Background:**

Computerized decision-support systems (CDSS) and performance-based incentives (PBIs) have potential to contribute to client satisfaction with health services. However, rigorous evidence is lacking on the effectiveness of these strategies in lower-income countries such as Ghana. This study aimed to determine the effect of a combined CDSS-PBI intervention on client satisfaction with maternal health services in primary facilities in the Upper East Region of Ghana.

**Methods:**

We employed a quasi-experimental controlled baseline and endline design to assess the effect of the combined interventions on client satisfaction with maternal health services, measured by quantitative pre/post-test client satisfaction survey. Our analysis used difference-in-difference logistic regression, controlling for potential covariates, to compare variables across intervention and comparison facilities at baseline and endline.

**Results:**

The combined CDSS-PBI intervention was associated with increased or unchanged client satisfaction with all maternal health services compared at endline. Antenatal client difference-in-difference of mean satisfaction scores were significant at endline for intervention (n = 378) and comparison (n = 362) healthcare facilities for overall satisfaction (DiD 0.058, p = 0.014), perception of providers’ technical performance (DiD = 0.142; p = 0.006), client-provider interaction (DiD = 0.152; p = 0.001), and provider availability (DiD = 0.173; p = 0.001). Delivery client difference-in-difference of satisfaction scores were significant at endline for intervention (n = 318) and comparison (n = 240) healthcare facilities for overall satisfaction with delivery services (DiD = 0.072; p = 0.02) and client-provider interaction (DiD = 0.146; p = 0.02). However, mean overall satisfaction actually reduced slightly in intervention facilities, while DiD for technical performance and provider availability were not significant.

**Conclusion:**

This combined CDSS-PBI intervention was associated with greater antenatal and delivery client satisfaction with some aspects of maternity services within two years of implementation. It could be expanded elsewhere if funds allow, though further research is still required to assess cost-effectiveness and long-term effects on client satisfaction and maternal health outcomes.

## Introduction

### Maternal health services, quality, and client satisfaction

Maternal and neonatal mortality statistics have improved globally, but remain poor. Globally, the annual neonatal mortality rate is approximately 18/1,000 live births, while the maternal mortality ratio is 216 deaths per 100,000 live births [1]. An estimated 99% of maternal deaths occur in low and middle-income countries (LMICs), more than half in sub-Saharan Africa [1]. In Ghana, the maternal mortality ratio is 319 deaths per 100,000 live births and the neonatal mortality rate is 25 deaths per 1,000 live births [2]. The 2014 Ghana Demographic and Health Survey estimated that 73% of births occurred in health facilities, with lower rates in poorer regions [3]. Similarly, skilled birth attendance rates differed between urban and rural areas in Ghana, averaging 74% and 43% respectively [4].

Poor uptake of maternal and neonatal health services contributes to these outcomes, implying some level of dissatisfaction among antenatal and postnatal clients [5]. Client satisfaction refers to how well women’s perceived healthcare needs and expectations are met [6, 7] and is an important component, along with provision quality at facilities (e.g. service readiness, adherence to guideline-based care), of antenatal and postnatal service quality [8–11] WHO’s definition of quality of care includes ‘people-centred’ (i.e. accounting for ‘preferences and aspirations of individual service-users’ and community cultures) with ‘safe, effective, timely, efficient, and equitable’ characteristics (12). Thus, the WHO framework for the quality of maternal and newborn healthcare links dimensions of provision and experience of care (12). Clients who are satisfied with their experience of maternal services are more likely to attend services and follow advice, contributing to better outcomes [12]. In LMICs such as Ghana, health system impediments affecting client satisfaction and care provision include insufficient competent and motivated health professionals, incorrect diagnoses and treatment, insufficient essential physical resources, and poor referral systems (10, 12, 14-[13]).

Improving satisfaction with maternal healthcare thus requires ensuring access to and provision of good-quality antenatal, delivery, and postnatal services along with effective communication, respect and dignity, and emotional support [10, 14]. Providers are key to client satisfaction with maternal healthcare, and if they provide poor or disrespectful care, clients are unlikely to be satisfied with services [15]. For example, Ghanaian antenatal and postnatal clients expressed dissatisfaction [16, 17] due to poor staff attitudes, long waiting times, insufficient healthcare staff, frequent hospital referrals, and lack of ambulances [18]. Quality assessment of 86 health facilities in former Brong-Ahafo region revealed that quality of routine and emergency intrapartum and postnatal care was generally low [10]. Nesbit et al described poor quality of care at Ghanaian health facilities using a service readiness assessment but did not assess perceived quality [10]. Diamond-Smith et al describe the disconnect between clinically-assessed and client perceived quality of care in Kenya and Namibia [13].

Several interventions have been implemented in Ghana to improve the quality of maternal health services and to reduce maternal deaths. This includes the 1995 National Safe Motherhood Programme, Community-based Planning and Services concept, a national approach to essential community-based health services provision involving health planning and service delivery with communities, targeting of deprived areas, and bringing health services closer to communities [19–23], and free antenatal care to all pregnant women [20, 24, 25]. Despite these, poor quality-of-care and dissatisfaction with maternal health services remain the main reason reported for services under usage. Poor maternal health staff attitudes reportedly discourage women, who instead prefer traditional birth attendants [26–28]. Thus, improving maternal health outcomes in Ghana requires improving women’s satisfaction with maternal health services [29–31].

### Computerised decision-support and performance-based incentives

Evidence suggests that computerized decision-support systems (CDSS) and performance-based incentives (PBIs) can improve health service provision and thus client satisfaction with care [32–40]. CDSS are designed to support clinical decisions during diagnostic or therapeutic care [41]. Common CDSS include drug-interaction checking, preventive care reminders, and adverse drug event detection [42–44]. CDSS, when well designed and used, can be effective tools for reducing the ‘know-do gap’ and enhancing quality-of-care, though they are not without challenges as shown in the literature [32, 45, 46]. An assessment of the effect of electronic medical records-based clinical decision-support on HIV care in resource-constrained settings reported reductions in documentation errors, missed appointments, missed CD4 results, and waiting times [47]. An Italian study reported that introducing CDSS was associated with a 16% reduction in the number of inappropriate laboratory tests requested, which also reduced costs [48, 49]. A cross-sectional study in northern Ghana showed that CDSS implementation was associated with reduced proportions of delivery complications and fewer maternal deaths [50]. Another study in northern Ghana indicated CDSS increased detection of pregnancy complications during antenatal care [32]. A study of the effects of CDSS on provider workflow in health facilities in northern Ghana showed history taking for women who had subsequent examinations increased from 58% to 95% [36].

PBIs refer to “*both monetary and non-monetary incentives to encourage health-related actions or achievement of performance targets*” [51], and can be supply-side and demand-side. Supply-side PBIs are used to improve the quality and availability of services through awards to staff for attaining organisational goals or thresholds related to health outcomes, service delivery, quality of care, or service use. Demand-side PBIs encourage use of essential health services through gifts to clients to change specified health-related behaviours [51]. Research suggests PBIs contribute to improvements in the provision of maternal health services and client satisfaction with care, though the literature also includes negative consequences [52–58]. For example, studies in Nigeria and Ghana showed that health-workers who received PBIs were more motivated to complete tasks than their counterparts who did not receive incentives [59]. A study of pay-for-performance (P4P) on use and quality of antenatal and delivery care in Rwanda showed it had a positive impact on the quality of maternal and child health services [60]. PBIs have been credited with improving antenatal, skilled birth attendance, and contraception coverage in Burundi [54, 61]. Although evidence suggests that PBIs can improve maternal and neonatal health, they had no significant effects on maternal or neonatal deaths suggesting information on how quality was measured was inadequate and further research needed [56, 62].

Despite significant evidence on the individual effects of CDSS and PBIs on provider performance and client satisfaction in LMICs, authors are not aware of research on the combined effects of CDSS and PBIs on client satisfaction. We found no published studies of the effects of a combined intervention on maternal health services satisfaction in LMICs. Additionally, existing studies focused on tertiary-level facilities rather than the primary-level facilities serving most antenatal and postnatal clients.

### Objectives

This study aimed to investigate the effect of a combined CDSS-PBI intervention on client satisfaction with maternal health services in twelve primary facilities in the Upper East Region of Ghana. Objectives were to assess: (i) combined effects of CDSS-PBI interventions on antenatal clients’ satisfaction with care; and (ii) combined effects of CDSS-PBI interventions on delivery clients’ satisfaction with care.

## Methods

### Study sites

The study was conducted in twelve health facilities in Kassena-Nankana (KND) and Builsa districts in the Upper East Region of Ghana. Maternal and child health services are considered particularly poor in the Upper East Region of Ghana, due to insufficient and poorly motivated staff, inadequate use of computers, poor access to reproductive health guidance, staff non-adherence to protocols, poor diagnosis, and inadequate referrals [4, 63–65]. Institutional MMR in the Upper East Region in 2010 was 352 per 100,000 live births. In Kassena-Nankana and Builsa districts in 2010, MMR was estimated at 367 and 259 per 100,000 live births respectively [66]. A cross-sectional study of 400 new mothers indicated 93% had delivered in a health facility and 97% had received antenatal care with 75% having four or more ANC visits [67]. An estimated 207 and 100 health-workers serve health facilities in KND and Builsa Districts, respectively. Nurses and midwives are responsible for the provision of maternal and neonatal health services in health facilities. In these districts in the Upper East Region of Ghana, inadequate health personnel, heavy workload and poor motivation (36, 52) challenge health facilities in the provision of care.

KND, the intervention district, covers a total approximate area of 1,675 square kilometres with an estimated population of 152,000 [68]. It is served by one hospital, located in the district capital Navrongo, six health centres, one private clinic, and twenty-seven Community-based Health Planning and Services (CHPS) compounds.

Builsa, the comparison district, covers a 2,220 square-kilometre area southwest of KND with an estimated population of 95,800. It is served by the district hospital, located in the district capital Sandema and serving as referral centre for all district health facilities. Additionally, Builsa has six main health centres and thirteen CHPS compounds.

### Study design

A quasi-experimental intervention study was conducted, with data collection at baseline and endline in 6 intervention and 6 comparison primary healthcare facilities in KND and Builsa districts respectively. This design was chosen because it can help establish causal impact of an intervention on a target population without random assignment [69].

#### Intervention

As part of the European Union funded Quality of maternal and neonatal health (QUALMAT) study in KND, CDSS and PBIs were implemented in six intervention facilities for two years from April 2012 to April 2014 [32, 36, 40]. The CDSS component consisted of computerized guidance and clinical support for antenatal and delivery care up to 24 hours after delivery. To improve maternal health services [64, 70], 35 purposively-selected maternal healthcare providers (i.e. midwives, community health nurses, health facility managers) completed six trainings on use of computer software to guide implementation of WHO *‘Pregnancy*, *Childbirth*, *Postpartum and Newborn Care*: *a guide for essential practice’* guidelines [71–73]. One laptop, with the CDSS installed and IT support included, was given per intervention facility for three trained midwives and nurses to share during the two-year study period [32, 36, 71]. The rest of the trained health staff were mangers tasked to supervise the use of the CDSS at the facility level.

The PBI component, rewarding best-performing midwives and facilities with domestic appliances (e.g. fans, stoves, blenders, freezers, fridges, television sets, blenders, saucepans, cloths, tea kettles, microwaves) and certificates of recognition [40], was implemented from July 2012 to March 2014 to improve CDSS users’ morale. Key performance indicators were defined through meetings between healthcare providers and regional and district health directorates. Performance indicators, focusing on process and outcomes, included proportion of ANC visits recorded in CDSS; proportion of pregnant women who received tetanus vaccination; proportion of pregnant women who received iron supplementation; proportion of pregnant women who received counselling for safe sex; proportion of pregnant women who received counselling for HIV; proportion of births attended by skilled personnel; proportion of partographs completed in CDSS; partograph usage rate; proportion of women referred based on CDSS recommendation; and newborn immunization coverage. Three awards ceremonies (held December 2012, September 2013, and February 2014) additionally acknowledged best-performing midwives and facilities. additional ongoing incentives included regular supervision, verbal appreciation, and provision of furniture and small monthly allowances [40, 64, 74].

#### Outcomes

Binary outcomes were client satisfaction (yes/no) with overall care and specifically with provider technical performance, interaction, and availability.

#### Sampling

Sample sizes were calculated to detect a difference of 10% in satisfaction between intervention and comparison arms and between pre and post intervention time-periods, with a two-sided test at a significance level of 5% and a power of 80%, for quality-of-care assuming a baseline of 60% and independence between interviews. This gave us a minimum total sample of 752 women, 376 in intervention facilities and 376 in comparison facilities for both antenatal and delivery objectives. Thus, interviewers aimed to include approximately 63 women per facility who attended an antenatal consultation and 63 per facility who recently gave birth. As we did not have the resources to follow client cohorts prospectively, all ANC and delivery clients visiting study facilities during baseline and endline data collection periods were eligible for inclusion.

### Data collection

Quantitative data were collected by client exit-survey interviews lasting approximately 15 minutes. Questions included socio-demographic characteristics, technical performance (i.e. reception of client, compassion, respect shown to client, adequacy of drugs and diagnosis), client-provider interaction, and provider availability–common components of client satisfaction used to assess health staff performance. The questionnaires for antenatal and delivery clients (S1 File) were developed based on similar tools in the literature [75–78] and expert advice, and pretested with 30 ANC clients at the War Memorial Hospital and 30 delivery clients at Sandema Hospital. After tool finalisation, baseline satisfaction survey data were collected in 2010 and endline in 2014.

Twelve research assistants were trained at the Navrongo Health Research Center for three days to conduct exit interviews with pregnant and postpartum women leaving health facilities after receiving maternal healthcare from providers. Data collectors attended facilities daily to conduct as many interviews as possible with antenatal and delivery clients. Most interviews in KND were conducted in Kassem and Nankam languages and in Builsa language in Builsa District. Written informed consent was obtained from all women before participation.

### Analysis

Data were entered in Microsoft Excel and transferred to Stata version 13 for analysis. Descriptive statistics were summarised, including age, weeks of gestation, parity, number of antenatal visits, educational level, and type of delivery. Antenatal and delivery care satisfaction response categories were recoded as binary (1 for satisfied, 0 for unsatisfied) from the original five categories, to reduce irrelevant detail and increase cell sizes.

Exploratory Factor Analysis was used for data reduction. Groups of variables were defined and mean scores calculated for all selected facilities in intervention and comparison areas, per variable and group (i.e. intervention or comparison). Principal Component Analysis was used to extract key factors, followed by a Varimax rotation with Kaiser normalisation. The number of factors retained was based on eigenvalues ≥1. Statistical analysis using difference in difference logistic regression has the potential to account for some initial differences that might have existed between the intervention and comparison health facilities. Controlling for some covariates was also appropriate.

Difference-in-Difference (DiD) analysis was performed, using a logit model and controlling for *a priori* confounders, to compare changes in outcomes between intervention and comparison groups over time (i.e. DiD results are based on differences in means between intervention and comparison groups at baseline compared to endline for each of the four outcome scores measured). Linear regression was used to estimate means, with p<0.05 used as the threshold for statistical significance. Potential confounders adjusted for in analyses as continuous variables were age, gravidity, gestational weeks, and number of ANC visits, while education level was categorical (i.e. never attended, attended primary, attended secondary, attended tertiary). Results were reported using significant digits (82).

### Ethics

Ethical approval was obtained from the Institutional Review Board of the Navrongo Health Research Centre, Ghana (reference NHRCIRB116). Written informed consent was obtained from all women before enrolment in the study. To guarantee confidentiality, study tools and outcomes did not include patient identifiers.

## Results

### Demographic characteristics

#### Antenatal clients

Table 1 shows demographic characteristics of antenatal participants at baseline (n = 709) and endline (n = 740). At baseline, 58% and 48% of intervention and comparison antenatal clients respectively were aged 20–29 years compared to 58% and 52% respectively at endline. At baseline, 44% and 49% of intervention and comparison clients respectively never attended school, compared to 38% and 54% respectively at endline. At baseline, mean gestational weeks were similar at 25.4 for intervention and 25.3 for comparison, shifting to 25.7 and 23.4 respectively at endline. At baseline, 33% and 23% of intervention and comparison clients respectively were primigravida, while 27% and 22% were on their second pregnancy at endline.

**Table 1:** Demographic characteristics of antenatal and delivery clients at baseline and endline.

Antenatal clients	Baseline		Endline	
Variable	Intervention n (%)	Comparison n (%)	Intervention n (%)	Comparison n (%)
**Total participants**	**341 (100)**	**368 (100)**	**378 (100)**	**362 (100)**
**Age**				
10–19	54 (15.8)	66 (17.9)	54 (14.2)	53 (14.6)
20–29	198 (58.1)	175 (47.6)	220 (58.2)	188 (51.6)
30–39	83 (24.3)	105 (28.4)	98 (25.9)	112 (30.8)
40–49	3 (0.9)	19 (5.1)	6 (1.6)	9 (2.5)
50+	1 (0.3)	0 (0.0)	0 (0.0)	0 (0.0)
Missing data	2 (0.6)	3 (0.8)	0 (0.0)	2 (0.5)
**Education**				
Never attended school	149 (43.7)	181 (49.2)	142 (37.6)	196 (53.8)
Primary/Junior High School	78 (22.9)	126 (34.2)	93 (24.6)	94 (25.8)
Senior High School	101 (29.6)	40 (10.9)	119 (31.5)	66 (18.1)
Tertiary	9 (2.6)	2 (0.5)	23 (6.1)	7 (1.9)
Missing data	4 (1.2)	19 (5.2)	1 (0.3)	1 (0.3)
**Mean gestational weeks (range)**	25.4 (5; 38)	25.3 (8; 39)	25.7 (6; 40)	23.4 (6; 40)
**Antenatal visits**				
Visit 1	57 (16.7)	78 (21.2)	82 (21.7)	138 (37.9)
Visit 2	79 (23.2)	85 (23.1)	65 (17.2)	66 (18.1)
Visit 3	47 (13.8)	73 (19.8)	53 (14.0)	47 (12.9)
Visit 4	64 (18.8)	51 (13.9)	42 (11.1)	51 (14.0)
Additional visits	87 (25.5)	80 (21.7)	136 (36.0)	61 (16.8)
Missing data	7 (2.1)	1 (0.3)	0 (0.0)	1 (0.3)
**Gravidity**				
Primigravida (1)	113 (33.1)	83 (22.6)	97 (25.7)	72 (19.8)
Multigravida (2)	79 (23.2)	75 (20.4)	101 (26.7)	80 (22.0)
Multigravida (3)	47 (13.8)	66 (17.9)	70 (18.5)	72 (19.8)
Multigravida (4)	56 (16.4)	71 (19.3)	50 (13.3)	69 (19.0)
Multigravida (5)	31 (9.1)	41 (11.2)	39 (10.3)	32 (8.9)
Grand multigravida (6+)	15 (4.4)	29 (7.9)	19 (5.0)	34 (9.3)
Missing data	0 (0.0)	3 (0.8)	2 (0.5)	5 (1.4)
**Delivery clients**	**Baseline**		**Endline**	
**Variable**	**Intervention n (%)**	**Comparison n (%)**	**Intervention n (%)**	**Comparison n (%)**
**Total participants**	**328 (100)**	**376 (100)**	**318 (100)**	**240 (100)**
**Age**				
10–19	52 (15.9)	59 (15.7)	61 (19.2)	39 (16.2)
20–29	180 (54.9)	201 (53.5)	183 (57.5)	135 (56.0)
30–39	79 (24.1)	105 (27.9)	63 (19.8)	56 (23.2)
40–49	12 (3.7)	11 (2.9)	9 (2.8)	10 (4.2)
50+	0 (0.0)	0 (0.0)	1 (0.3)	0 (0.0)
Missing data	5 (1.52)	0 (0.0)	1 (0.3)	1 (0.4)
**Educational level**				
Never attended school	143 (43.6)	174 (46.3)	124 (39.0)	98 (40.7)
Primary/Junior High School	88 (26.8)	121 (32.2)	78 (24.5)	82 (34.0)
Senior High School	91 (27.7)	74 (19.7)	104 (32.7)	56 (23.2)
Tertiary	5 (1.5)	5 (1.3)	4 (1.3)	3 (1.2)
Missing data	1 (0.3)	2 (0.5)	8 (2.5)	2 (0.8)
**Mean days at facility (range)**	4.2 (1; 48)	7.7 (1; 28)	1.1 (1; 5)	1.2 (1; 5)
**Neonatal health at delivery**				
Good health	318 (97.0)	369 (98.1)	310 (97.5)	236 (97.9)
Health concerns at birth	6 (1.8)	0 (0.0)	7 (2.2)	2 (0.8)
Stillbirth	1 (0.3)	1 (0.3)	1 (0.3)	0 (0.0)
Missing data	3 (0.9)	6 (1.6)	0 (0.0)	3 (1.2)
**Mean parity (range)**	2.6 (1; 10)	2.6 (1; 9)	2.8 (1; 16)	2.7 (1; 8)
**Type of delivery**				
Planned caesarean-section	0 (0.0)	2 (0.5)	1 (0.3)	3 (1.2)
Emergency caesarean-section	4 (1.2)	9 (2.4)	1 (0.3)	0 (0.0)
Vaginal (assisted)	4 (1.2)	4 (1.1)	2 (0.6)	4 (1.7)
Vaginal (normal)	318 (97.0)	339 (95.5)	314 (98.7)	234 (97.1)
Missing data	2 (0.6)	2 (0.5)	0 (0.0)	0 (0.0)

#### Delivery clients

704 delivery clients participated at baseline and 558 at endline. At baseline, 55% and 54% of intervention and comparison delivery clients respectively were aged 20–29 years compared to 58% and 56% respectively at endline. At baseline, 44% and 47% of intervention and comparison delivery clients respectively never attended school, compared to 39% and 41% respectively at endline. At baseline, intervention and comparison delivery clients spent a mean of 4.2 days and 7.7 days respectively at facilities for delivery, compared to a mean of 1.1 days and 1.2 days respectively at endline. At baseline, 2% and 0% of intervention and comparison delivery clients respectively reported neonatal health issues at birth compared to 2% and 1% respectively at endline. At baseline, 1.2% and 2.4% of intervention and comparison delivery clients respectively had an emergency caesarean-section compared to 0.3% and 1.7% respectively at endline (Table 1).

### Adjusted effects of the CDSS-PBI intervention on client satisfaction

#### Antenatal clients

Table 2 shows multivariable logistic regression analysis of client satisfaction with antenatal and delivery services, comparing the difference-in-difference of mean numbers of satisfied clients in intervention and comparison areas at baseline and endline, indicating improved satisfaction in three of four elements measured. In total, 709 antenatal clients (341 intervention, 368 comparison) were included in baseline analyses and 740 (378 intervention, 362 comparison) in endline analyses. At baseline, the mean number of antenatal clients reporting satisfaction with provider technical performance was similar at 0.560 and 0.580 (p = 0.59) in intervention and comparison areas respectively, compared to significantly higher mean satisfaction in comparison areas (0.521 versus 0.643 respectively; p = 0.001) at endline. The difference-in-difference of 0.142, statistically significant at p = 0.006, showed a significantly greater increase in mean satisfaction with technical performance in comparison than in intervention facilities at endline. At baseline, the mean number of clients reporting satisfactory interactions with providers was similar 0.648 and 0.607 (p = 0.21) in intervention and comparison areas respectively, compared to significantly higher satisfaction in intervention areas (0.889 versus 0.696 respectively; p<0.001) at endline. The difference-in-difference of 0.152, significant at p = 0.001, showed a significantly greater increase in satisfaction with provider interaction in intervention than in comparison facilities at endline. At baseline, the mean number of clients reporting satisfactory provider availability was similar at 0.592 and 0.634 (p = 0.25) in intervention and comparison areas respectively, compared to significantly higher mean satisfaction in intervention areas (0.688 versus 0.556 respectively; p<0.001) at endline. The difference-in-difference of 0.173, significant at p = 0.001, showed a significantly greater increase in satisfaction with provider availability in intervention than in comparison facilities at endline. At baseline, the mean number of clients reporting general satisfaction was significantly lower in intervention than comparison areas (0.919 and 0.965 respectively; p = 0.006), compared to increased satisfaction in intervention facilities and reduced satisfaction in comparison facilities (0.947 versus 0.959 respectively) at endline, though the difference in means of 0.012 was not significant at p = 0.47. However, the difference-in-difference of 0.058 was significant at p = 0.014, showing a significantly greater increase in mean overall satisfaction in intervention than in comparison facilities at endline.

**Table 2:** Multivariable logistic regression of antenatal and delivery client satisfaction with services, comparing intervention to comparison at baseline and endline.

**Antenatal outcomes**	**Baseline**				**Endline**				**DiD**	
	**Intervention (n 341)**	**Comparison (n 368)**	**Diff**	**P-value**	**Intervention (n 378)**	**Comparison (n 362)**	**Diff**	**P-value**	**Diff-Diff**	**P-value**
	**Mean**	**Mean**			**Mean**	**Mean**				
Technical Performance	0.560	0.580	0.020	0.592	0.521	0.643	0.122	0.001	0.142	0.006
Client-provider interaction	0.648	0.607	0.041	0.214	0.889	0.696	0.193	0.000	0.152	0.001
Provider availability	0.592	0.634	0.042	0.251	0.688	0.556	0.132	0.000	0.173	0.001
General satisfaction	0.919	0.965	0.046	0.006	0.947	0.959	0.012	0.472	0.058	0.014
**Delivery outcomes**	**Baseline**				**Endline**				**DiD**	
	**Intervention (n 325)**	**Comparison (n 379)**	**Diff**	**P-value**	**Intervention (n 318)**	**Comparison (n 240)**	**Diff**	**P-value**	**Diff-Diff**	**P-value**
	**Mean**	**Mean**			**Mean**	**Mean**				
Technical Performance	0.979	0.864	0.115	0.000	0.966	0.885	0.081	0.074	0.034	0.528
Client-provider interaction	0.767	0.792	0.025	0.463	0.994	0.872	0.122	0.017	0.146	0.017
Provider availability	0.653	0.716	0.064	0.097	0.378	0.432	0.054	0.347	0.118	0.088
General satisfaction	0.990	0.919	0.071	0.000	0.980	0.980	0.001	0.000	0.072	0.017

NB: Potential confounders included in analysis were age, education level, days at facility, gestation weeks, gravidity (number of pregnancies), and parity (number of deliveries).

#### Delivery clients

In total, 704 delivery clients (328 intervention, 376 comparison) were included in baseline analyses and 558 (318 intervention, 240 comparison) in endline analyses. Table 2 shows improved satisfaction in one of four elements measured (i.e. client-provider interactions). At baseline, the mean number of clients reporting satisfactory interactions with providers was similar at 0.767 and 0.792 (p = 0.46) in intervention and comparison areas respectively, compared to significantly higher satisfaction in intervention areas (0.994 versus 0.872 respectively; p = 0.02) at endline. The difference-in-difference of 0.146, significant at p = 0.01, showed a significantly greater increase in satisfaction with provider interactions in intervention than in comparison facilities at endline. However, overall satisfaction changed quite differently. At baseline, the mean number of delivery clients reporting general satisfaction was significantly higher in intervention than comparison areas (0.990 versus 0.919 respectively; p<0.001), compared with lower satisfaction in intervention facilities and higher in comparison facilities (0.980 versus 0.980 respectively) at endline, with small but a significant difference in means of 0.001 (p<0.001). The difference-in-difference of 0.072, also significant at p = 0.02, showed a slight but significantly greater increase in overall satisfaction in comparison facilities versus intervention facilities at endline (Table 2).

Other delivery outcomes in Table 2 did not show significant changes. At baseline, the mean number of delivery clients reporting satisfaction with provider technical performance was significantly higher in intervention than comparison areas (0.979 and 0.864 respectively; p<0.001), compared to reduced satisfaction in intervention facilities and increased satisfaction in comparison facilities (0.966 and 0.885 respectively) at endline, though the difference in means of 0.081 was not significant at p = 0.07. The difference-in-difference was also not significant (p = 0.53). At baseline, the mean number of clients reporting satisfaction with provider availability was similar at 0.653 and 0.716 (p = 0.10) in intervention and comparison areas respectively, compared to lower satisfaction in both (0.378 versus 0.432 respectively) at endline, though the difference in means of 0.054 was not significant at p = 0.35. Similarly, the difference-in-difference was not significant (p = 0.09).

## Discussion

### Primary findings

The primary contribution of this study is in showing that a combined CDSS and PBI intervention can improve maternity clients’ satisfaction in a resource-constrained setting. Results indicate that the combined intervention was associated with increased client satisfaction with elements of maternal health services, though this was not consistent across all elements or types of clients. Among antenatal clients, the intervention was associated with significantly increased satisfaction in three of the four elements measured, namely provider interactions, provider availability, and overall satisfaction. Among delivery clients, however, the intervention was associated with significantly increased satisfaction in only one of the four elements, namely provider interactions. It remains unclear why delivery clients in intervention facilities expressed reduced satisfaction at endline and why delivery clients appeared less satisfied than antenatal clients across multiple elements. However, anecdotal evidence suggested women avoided hospital delivery in northern Ghana due to expectations of poor treatment, while Adjei *et al* found that women with middle-school education or above had half the odds of being satisfied with delivery services than women with no education [79]. Among both client types in this study, the intervention was associated with somewhat decreased satisfaction with provider technical performance, suggesting it either did not improve technical performance or created excessive expectations among clients. Improvements in satisfaction in comparison groups may also relate to parallel maternal and child health initiatives implemented by other projects during this intervention that likely reduced observable differences between intervention and comparison groups [20].

Further facility audits or research data are needed to determine some differences found, for example why endline emergency caesarean-section rates were so low in both intervention and comparison facilities (i.e. 0.3% and 0% respectively, while WHO recommends 10–15%) [80]. Similarly, there was no clear explanation why delivery clients appeared to have much longer average facility stays at baseline versus endline (i.e. 6 days versus 1 day respectively). Thirteen clients with lengthy stays of over three weeks caused this high baseline mean (i.e. 7 in intervention, 6 in comparison facilities), though this did not explicitly relate to worse health outcomes and may instead have related to socioeconomic circumstances for which data were not collected (e.g. distance from or problems at home).

Authors found no published studies examining the combined effects of CDSS and PBI interventions on client satisfaction with maternal health services. However, individual effects of these two interventions on client satisfaction have been reported globally. For example, a study on whether CDSS could increase healthcare use and quality in primary facilities in India showed it was associated with significant improvements in a Global Patient Assessment of Care Index (mean DiD 7.9, p<0.001), with largest gains made in patient communication, technical quality, and general satisfaction with care [81]. A Bangladesh study of the effects of PBI on maternal health services quality showed facility quality scores increased from 55% to 78% during the 14-month intervention with significant improvements in antenatal care, postnatal counselling, institutional delivery, and client satisfaction [82]. Global evidence indicates that in addition to client satisfaction, routine usage of CDSS has potential to improve maternal health outcomes, including reducing maternal mortality [36, 50]. Similarly, though evidence is not as robust, PBIs can improve motivation, technology usage, and work performance among health-workers contributing to improved maternal outcomes [51].

### Implications

Findings highlight the potential of a combination of CDSS and PBI interventions to improve health service-users satisfaction in a resource-constrained setting such as northern Ghana. Evidence from other settings supports the need for health-workers using CDSS for patient care to receive intensive training [83]. Thus, findings could have national implications, as policy-makers look for innovative ways to improve midwives performance so as to achieve Sustainable Development Goal 3.

Further research is still needed to determine whether CDSS and PBI interventions are sustainable either together or separately, more cost-effective together or as individual components, and can yield similar or better gains in primary facilities than in other settings. Further qualitative research is needed to interpret the meaning of antenatal and delivery service satisfaction in this and other resource-constrained settings. For example, on how reported satisfaction was influenced by women’s awareness of additional CDSS equipment or efforts made by staff to achieve PBIs, as compared to the intended contributory effects of CDSS or PBIs on staff performance per se. However, in the meantime, these results can help inform national and regional decision-makers and policy influencers in their support of the Ministry of Health and Ghana Health Service (GHS) to strengthen maternal health services provision within WHO standards for improving quality of maternal and newborn care in health facilities (12). Collaborative development of national and sub-national health-worker incentives and decision-support policies, or policy components within broader strategic planning, is likely to have a positive influence. For example, MOH and GHS could introduce policy on the use of CDSS by nurses and midwives to facilitate adherence to WHO reproductive health guidelines. Such guidance should use lessons from this intervention to motivate midwives and other staff to improve health service quality in Ghana.

### Limitations

As there is no empirical evidence on the combined effects of CDSS and PBI strategies on client satisfaction, this study helps fill this gap. However, study limitations must be considered. First, clients in both intervention and comparison facilities reporting improved general satisfaction could have been because study districts were in the Navrongo Health Research Center catchment area and thus exposed to other maternal and child health initiatives (e.g. the Ghana Essential Health Study to improve maternal and newborn care implemented in Builsa District) that could have improved overall satisfaction [20]. However, these external influences would have affected intervention and comparison facilities similarly and not the inter-group differences found. Relatedly, there may have been some sampling bias, due to differing endline sample sizes as surveyors had more difficulty identifying eligible women in comparison facilities. Second, this study focused on maternal healthcare clients rather than the general healthcare client population in KND and Builsa districts. However, future research could examine the effects of CDSS and PBI on satisfaction among other sub-sets or all health service clients. Third, quantitative research cannot provide insight into how interventions affect outcomes and qualitative research is needed to provide additional nuance. Finally, a facility *readiness and adherence to guideline-based care* assessment would have provided useful information on overall quality-of-care but was not done (12).

## Conclusions

This combined CDSS-PBI intervention significantly improved elements of antenatal and delivery client satisfaction with maternal health services within two years of implementation in resource-constrained facilities in northern Ghana. This shows promise for other settings, though further research is still required to assess cost-effectiveness and sustainability, including the long-term effects of these interventions on client satisfaction and maternal health outcomes.

## Supporting information

S1 File (DOCX)
