# Supplementary material for: Effects of combined decision-support and performance-based incentives on reported client satisfaction with maternal health services in primary facilities: A quasi-experimental study in the Upper East Region of Ghana
Source: PLoS One. 2021 Apr 20;16(4):e0249778. doi: 10.1371/journal.pone.0249778 (PMC8057590; doi:10.1371/journal.pone.0249778)
Supplement: S1 File — (DOCX) [file pone.0249778.s001.docx]

# S1 File

# Questionnaire for women’s satisfaction survey on antenatal care

**Country: Ghana**

| **Administrative** **Information** |
| --- |

| **Facility Information** | | |
| --- | --- | --- |
| A.1 | Name health facility |  |
| A.2 | Name district |  |
| **Information about interview** | | |
| A.3 | Name interviewer |  |
| A.4 | Date interview | _ _ / _ _ / _ _ _ _ |
| A.5 | **Time interview started** | **h m** |
| **Information about pregnant woman** | | |
| A.6 | Age |  |
| A.7 | Week of gestation | __________ weeks |
| A.8 | Nr of ANC visit | ᴑ Visit 1  ᴑ Visit 2  ᴑ Visit 3  ᴑ Visit 4  ᴑ Other, specify: ________ |
| A.9 | Reason of ANC visit | ᴑ Visit for routine ANC  ᴑ Visit due to symptoms or disease |
| A.10 | Was this a | ᴑ Scheduled visit  ᴑ Unscheduled visit |
| A.11 | Number of pregnancies (including present pregnancy) |  |
| A.12 | Literate | Can read: ᴑ Yes ᴑ No  Can write: ᴑ Yes ᴑ No |
| A.13 | Education level | ᴑ Never attended school  ᴑ Primary school started  ᴑ Primary school finished  ᴑ Vocational training started  ᴑ Vocational training finished  ᴑ Secondary school started  ᴑ Secondary school finished  ᴑ Higher education started  ᴑ Higher education finishes |

| Inform patient that: The study looks at management of ANC  The study is completely confidential  No name is written on this questionnaire  She can refuse to answer individual questions if she wishes  The interview will take less than 15 minutes | | | | | |
| --- | --- | --- | --- | --- | --- |
| **Now I’m going to ask you some questions about the antenatal visit you just attended.** | | | | | |
| 1 | Are you satisfied with the reception you received at the health facility?  *(read out the options)* | | | 1 = very satisfied  2 = satisfied  3 = neutral  4 = unsatisfied  5 = very unsatisfied | |
| 2 | Are you satisfied with the level of privacy provided during the consultation?  *(read out the options)* | | | 1 = very satisfied  2 = satisfied  3 = neutral  4 = unsatisfied  5 = very unsatisfied | |
| 3 | Are you satisfied with the level of respect you received from the health worker?  *(read out the options)* | | | 1 = very satisfied  2 = satisfied  3 = neutral  4 = unsatisfied  5 = very unsatisfied | |
| 4 | Are you satisfied with the level of support you received from the health worker?  *(read out the options)* | | | 1 = very satisfied  2 = satisfied  3 = neutral  4 = unsatisfied  5 = very unsatisfied | |
| 5 | Are you satisfied about not having had to wait too long before seeing a health worker?  *(read out the options)* | | | 1 = very satisfied  2 = satisfied  3 = neutral  4 = unsatisfied  5 = very unsatisfied | |
| 6 | Did you have enough time with the health worker, or would you prefer?  *(read out the options)* | | | 1 = a lot more time  2 = a little more time  3 = time is about right  4 = less time | |
| 7 | Was the information you received about looking after your own health:  *(read out the options)* | | | 1 = not enough  2 = as much as you wanted  3 = too much  4 = no information received  5 = don’t remember | |
| 8 | Was the information you received about any tests (e.g. blood, urine) during pregnancy:  *(read out the options)* | | | 1 = not enough  2 = as much as you wanted  3 = too much  4 = no information received  5 = don’t remember | |
| 9 | Was the information you received about any treatment you might need during this pregnancy:  *(read out the options)* | | | 1 = not enough  2 = as much as you wanted  3 = too much  4 = no information received  5 = don’t remember | |
| 10 | Was the information you received about labour:  *(read out the options)* | | | 1 = not enough  2 = as much as you wanted  3 = too much  4 = no information received  5 = don’t remember | |
| 11 | Was the information you received about breastfeeding:  *(read out the options)* | | | 1 = not enough  2 = as much as you wanted  3 = too much  4 = no information received  5 = don’t remember | |
| 12 | Do you know danger signs that can occur during pregnancy?  *(read out the options)* | | | 1 = yes  2 = no | |
|  | **If the answer on 12 is ‘yes’ go to 13**  **If the answer on 12 is ‘no’ go to 14** | | | | |
| 13 | Can you mention some of these danger signs?  *(****do not*** *read the options, tick the signs the woman mentions)* | | ᴑ haemorrhage  ᴑ rupture of membranes  ᴑ dizziness and fainting  ᴑ fever  ᴑ premature contractions  ᴑ headache and blurred vision  ᴑ swelling of fingers, face and/or legs  ᴑ severe abdominal pain  ᴑ fast or difficult breathing  ᴑ convulsions  ᴑ others, specify: __________________ | | |
| 14 | Were you told how to recognise and proceed about some serious problems that can happen in pregnancy  *(read out the problem and ask for each problem if it was explained how to recognise and how to proceed)* | | | | |
|  |  | Told how to recognise | | | Told how to proceed |
|  | vaginal bleeding | 1 = yes 2 = no | | | 1 = yes 2 = no |
|  | rupture of membranes | 1 = yes 2 = no | | | 1 = yes 2 = no |
|  | fever | 1 = yes 2 = no | | | 1 = yes 2 = no |
|  | swelling of fingers, face and/or legs | 1 = yes 2 = no | | | 1 = yes 2 = no |
|  | premature contractions | 1 = yes 2 = no | | | 1 = yes 2 = no |
|  | dizziness and fainting | 1 = yes 2 = no | | | 1 = yes 2 = no |
|  | headache and blurred vision | 1 = yes 2 = no | | | 1 = yes 2 = no |
|  | convulsions | 1 = yes 2 = no | | | 1 = yes 2 = no |
|  | fast or difficult breathing | 1 = yes 2 = no | | | 1 = yes 2 = no |
|  | severe abdominal pain | 1 = yes 2 = no | | | 1 = yes 2 = no |
| 15 | Are you satisfied about the clinical examination done by the health worker?  *(read out the options)* | | | 1 = very satisfied  2 = satisfied  3 = neutral  4 = unsatisfied  5 = very unsatisfied | |
| 16 | Are you satisfied with the treatment and/or advice you received from the health worker?  *(read out the options)* | | | 1 = very satisfied  2 = satisfied  3 = neutral  4 = unsatisfied  5 = very unsatisfied | |
| 17 | Are you satisfied with the drugs prescribed by the health worker?  *(read out the options)* | | | 1 = very satisfied  2 = satisfied  3 = neutral  4 = unsatisfied  5 = very unsatisfied  6 = no drugs prescribed | |
| 18 | Are you satisfied about the level of knowledge of the health worker?  *(read out the options)* | | | 1 = very satisfied  2 = satisfied  3 = neutral  4 = unsatisfied  5 = very unsatisfied | |
| 19 | Are you satisfied about the attitude of the health worker?  *(read out the options)* | | | 1 = very satisfied  2 = satisfied  3 = neutral  4 = unsatisfied  5 = very unsatisfied | |
| 20 | Are you satisfied with the opportunity you received to ask questions?  *(read out the options)* | | | 1 = very satisfied  2 = satisfied  3 = neutral  4 = unsatisfied  5 = very unsatisfied | |
| 21 | Are you satisfied with the opportunity you received to express your concerns?  *(read out the options)* | | | 1 = very satisfied  2 = satisfied  3 = neutral  4 = unsatisfied  5 = very unsatisfied | |
| 22 | Are you satisfied about the drugs you received at the health facility?  *(read out the options)* | | | 1 = very satisfied  2 = satisfied  3 = neutral  4 = unsatisfied  5 = very unsatisfied  6 = did not receive any drugs | |
| 23 | Are you satisfied about the medical equipment available at the health facility?  *(read out the options)* | | | 1 = very satisfied  2 = satisfied  3 = neutral  4 = unsatisfied  5 = very unsatisfied | |
| 24 | Are you satisfied about the hygiene at the health facility?  *(read out the options)* | | | 1 = very satisfied  2 = satisfied  3 = neutral  4 = unsatisfied  5 = very unsatisfied | |
|  | **Finally, three questions to sum up** | | | | |
| 25 | If you get pregnant again will you come back to this health facility?  *(read out the options)* | | | 1 = yes  2 = no  3 = don’t know  *(always ask)*  Why? __________________________  __________________________ | |
| 26 | Would you recommend this health facility to a relative or friend for their antenatal checkups?  *(read out the options)* | | | 1 = yes  2 = no  3 = don’t know | |
| 27 | In general, how satisfied are you with the antenatal care you received during the consultation just finished?  *(read out the options)* | | | 1 = very satisfied  2 = satisfied  3 = neutral  4 = unsatisfied  5 = very unsatisfied | |

**Time interview ended: h m**

***Comments:***

# Questionnaire for women’s satisfaction survey on delivery care

**Country: Ghana**

| **Administrative** **Information** |
| --- |

| **Facility Information** | | |
| --- | --- | --- |
| A.1 | Name health facility |  |
| A.2 | Name district |  |
| **Information about interview** | | |
| A.3 | Name interviewer |  |
| A.4 | Date interview | _ _ / _ _ / _ _ _ _ |
| A.5 | **Time interview started** | **h m** |
| **Information about the interviewed woman** | | |
| A.6 | Age |  |
| A.7 | Number of deliveries (including this delivery) |  |
| A.8 | Literate | Can read: ᴑ Yes ᴑ No  Can write: ᴑ Yes ᴑ No |
| A.9 | Education level | ᴑ Never attended school  ᴑ Primary school started  ᴑ Primary school finished  ᴑ Vocational training started  ᴑ Vocational training finished  ᴑ Secondary school started  ᴑ Secondary school finished  ᴑ Higher education started  ᴑ Higher education finishes |
| **Information about the delivery** | | |
| A.10 | Kind of delivery | ᴑ vaginal delivery – normal  ᴑ vaginal delivery – assisted by equipment (vacuum or forceps)  ᴑ a planned caesarean delivery  ᴑ an emergency caesarean delivery |
| A.11 | Information on the newborn | ᴑ in good health at the time of the interview  ᴑ health problems at the time of the interview  ᴑ stillbirth  ᴑ child died after the delivery (neonatal death)  If child died: When; how many days after the delivery?  ________ days. |

| Inform patient that: The study looks at management of delivery  The study is completely confidential  No name is written on this questionnaire  She can refuse to answer individual questions if they wish  The interview will take less than 15 minutes | | | |
| --- | --- | --- | --- |
| **Now I’m going to ask you some questions about your delivery in the health facility.** | | | |
| 1 | Are you satisfied with the reception you received at the health facility?  *(read out the options)* | | 1 = very satisfied  2 = satisfied  3 = neutral  4 = unsatisfied  5 = very unsatisfied |
| 2 | Are you satisfied with the level of privacy provided during your stay in the health facility and the delivery?  *(read out the options)* | | 1 = very satisfied  2 = satisfied  3 = neutral  4 = unsatisfied  5 = very unsatisfied |
| 3 | Are you satisfied with the level of respect you received from the health worker(s)?  *(read out the options)* | | 1 = very satisfied  2 = satisfied  3 = neutral  4 = unsatisfied  5 = very unsatisfied |
| 4 | Are you satisfied with the level of support you received from the health worker(s)?  *(read out the options)* | | 1 = very satisfied  2 = satisfied  3 = neutral  4 = unsatisfied  5 = very unsatisfied |
| 5 | Are you satisfied with the information you received from the health worker(s) during labour, delivery and after the delivery?  *(read out the options)* | | 1 = very satisfied  2 = satisfied  3 = neutral  4 = unsatisfied  5 = very unsatisfied |
| 6 | Was the time you had to wait between arriving at the health facility and seeing a health worker;  *(read out the options)* | | 1 = far too long  2 = long  3 = neutral  4 = I was helped immediately; I  did not had to wait. |
| 7 | Was the time the health worker(s) spend with you during labour and delivery;  *(read out the options)* | | 1 = not enough  2 = enough  3 = neutral  4 = there was all the time a  health worker with me |
| 8 | How long (hours and if applicable days) did you spend in the health facility after you delivered? _________hours (__________ days) | | |
| 9 | Was the time you spent in the health facility after you delivered;  *(read out the options)* | | 1 = not long enough  2 = neutral (=enough)  3 = too long |
|  | Are you satisfied with the information you received about: | |  |
| 10 |  | - Breastfeeding   *(read out the options)* | 1 = very satisfied  2 = satisfied  3 = neutral  4 = unsatisfied  5 = very unsatisfied  6 = I did not received any  information on this topic |
| 11 |  | - Your own nutrition   *(read out the options)* | 1 = very satisfied  2 = satisfied  3 = neutral  4 = unsatisfied  5 = very unsatisfied  6 = I did not received any  information on this topic |
| 12 |  | - Postpartum care and hygiene   *(read out the options)* | 1 = very satisfied  2 = satisfied  3 = neutral  4 = unsatisfied  5 = very unsatisfied  6 = I did not received any  information on this topic |
| 13 |  | - Family planning   *(read out the options)* | 1 = very satisfied  2 = satisfied  3 = neutral  4 = unsatisfied  5 = very unsatisfied  6 = I did not received any  information on this topic |
| 14 |  | - Danger signs after childbirth for the mother - when you have to seek care again at a health facility   *(read out the options)* | 1 = very satisfied  2 = satisfied  3 = neutral  4 = unsatisfied  5 = very unsatisfied  6 = I did not received any  information on this topic |
| 15 |  | Can you give/mention some of these danger signs?  *(****do not*** *read the options, tick the signs the woman mentions)* | ᴑ vaginal bleeding  ᴑ convulsions  ᴑ fast and difficult breathing  ᴑ fever and too weak to get out  of bed  ᴑ severe abdominal pain  ᴑ fever  ᴑ breast swollen, red or tender  breasts, or sore nipple  ᴑ urine dribbling or pain on  micturition  ᴑ others: _____________ |
| 16 |  | - Danger signs after childbirth for the child - when you have to seek care again at a health facility   *(read out the options)* | 1 = very satisfied  2 = satisfied  3 = neutral  4 = unsatisfied  5 = very unsatisfied  6 = I did not received any  information on this topic |
| 17 |  | Can you give/mention some of these danger signs?  *(****do not*** *read the options, tick the signs the woman mentions)* | ᴑ difficulty in breathing  ᴑ convulsions  ᴑ fever  ᴑ feels cold  ᴑ diarrhoea  ᴑ difficulty in feeding  ᴑ no feeding at all  ᴑ pus from eyes  ᴑ skin pustules  ᴑ yellow skin  ᴑ a cord stump which is red or  draining pus |
| 18 | Are you satisfied about the clinical examination performed by the health worker(s) during labour and after the delivery?  *(read out the options)* | | 1 = very satisfied  2 = satisfied  3 = neutral  4 = unsatisfied  5 = very unsatisfied |
| 19 | Are you satisfied with the level of assistance provided by the health worker(s) during the delivery?  *(read out the options)* | | 1 = very satisfied  2 = satisfied  3 = neutral  4 = unsatisfied  5 = very unsatisfied |
| 20 | Are you satisfied with what was done to relief your pain during labour and delivery?  *(read out the options)* | | 1 = very satisfied  2 = satisfied  3 = neutral  4 = unsatisfied  5 = very unsatisfied  6 = nothing was done |
| 21 | Are you satisfied with the level of attention and care given to your newborn baby after delivery?  *(read out the options)* | | 1 = very satisfied  2 = satisfied  3 = neutral  4 = unsatisfied  5 = very unsatisfied |
| 22 | Are you satisfied with the level of attention and care given to you after delivery?  *(read out the options)* | | 1 = very satisfied  2 = satisfied  3 = neutral  4 = unsatisfied  5 = very unsatisfied |
| 23 | Are you satisfied with the help received for breastfeeding?  *(read out the options)* | | 1 = very satisfied  2 = satisfied  3 = neutral  4 = unsatisfied  5 = very unsatisfied  6 = no help received |
| 24 | Are you satisfied with the drugs prescribed by the health worker(s)?  *(read out the options)* | | 1 = very satisfied  2 = satisfied  3 = neutral  4 = unsatisfied  5 = very unsatisfied  6 = no drugs prescribed |
| 25 | Are you satisfied about the level of knowledge of the health worker(s)?  *(read out the options)* | | 1 = very satisfied  2 = satisfied  3 = neutral  4 = unsatisfied  5 = very unsatisfied |
| 26 | Are you satisfied about the attitude of the health worker(s)?  *(read out the options)* | | 1 = very satisfied  2 = satisfied  3 = neutral  4 = unsatisfied  5 = very unsatisfied |
| 27 | Are you satisfied with the opportunity you received to ask questions?  *(read out the options)* | | 1 = very satisfied  2 = satisfied  3 = neutral  4 = unsatisfied  5 = very unsatisfied |
| 28 | Are you satisfied with the opportunity you received to express your concerns?  *(read out the options)* | | 1 = very satisfied  2 = satisfied  3 = neutral  4 = unsatisfied  5 = very unsatisfied |
| 29 | Are you satisfied about the drugs you received at the health facility?  *(read out the options)* | | 1 = very satisfied  2 = satisfied  3 = neutral  4 = unsatisfied  5 = very unsatisfied  6 = did not receive any drugs |
| 30 | Are you satisfied about the medical equipment available at the health facility?  *(read out the options)* | | 1 = very satisfied  2 = satisfied  3 = neutral  4 = unsatisfied  5 = very unsatisfied |
| 31 | Are you satisfied about the hygiene at the health facility?  *(read out the options)* | | 1 = very satisfied  2 = satisfied  3 = neutral  4 = unsatisfied  5 = very unsatisfied |
|  | **Finally, three questions to sum up** | | |
| 32 | If you have to deliver again will you come back to this health facility?  *(read out the options)* | | 1 = yes  2 = no  3 = don’t know  *(always ask)*  Why? __________________  _______________________ |
| 33 | Would you recommend this health facility to a relative or friend for them to deliver?  *(read out the options)* | | 1 = yes  2 = no  3 = don’t know |
| 34 | In general, how satisfied are you with the care you received during your delivery at this health facility?  *(read out the options)* | | 1 = very satisfied  2 = satisfied  3 = neutral  4 = unsatisfied  5 = very unsatisfied |

**Time interview ended: h m**

***Comments:***
